# P-2235. Implementation of a Host-Protein Test (MMBV) Into Routine Care for Children Hospitalized with Symptoms of Lower Respiratory Tract Infection

**DOI:** 10.1093/ofid/ofae631.2388

**Published:** 2025-01-29

**Authors:** Vered Nir, Vered Schichter Konfino, Naama Kuchinski Cohen, Esther Levy, Maanit Shapira, Michal Stein, Adi Klein

**Affiliations:** Hillel Yaffe Medical Center, Hadera, Hefa, Israel; Hillel Yaffe Medical Center, Hadera, Hefa, Israel; Hillel Yaffe Medical Center, Hadera, Hefa, Israel; Hillel Yaffe Medical Center, Hadera, Hefa, Israel; Hillel Yaffe Medical Center, Hadera, Hefa, Israel; Sheba Medical Center, Tel HaShomer, HaMerkaz, Israel; Hillel Yaffe Medical Center, Hadera, Hefa, Israel

## Abstract

**Background:**

Infectious etiology is often unclear in children hospitalized with symptoms of lower respiratory tract infection (LRTI), driving antibiotic misuse. A host-protein test (MMBV) exhibits high diagnostic accuracy for differentiating bacterial from viral etiology. Best practices for test implementation should be established. We evaluate the effectiveness of implementing MMBV into routine care without specific antimicrobial stewardship (AMS) education by assessing impact on antibiotic administration to children hospitalized with LRTI symptoms.

Patient flow

**Figure:**
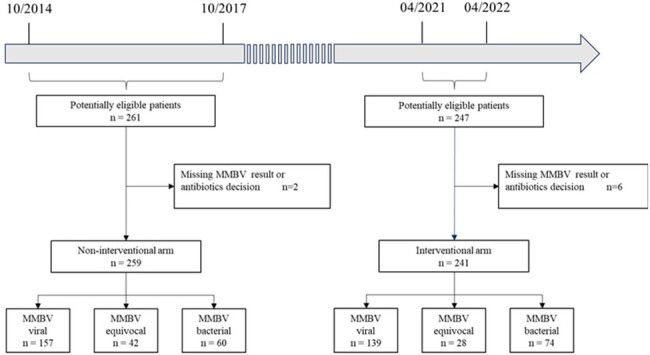

Eligibility required age 3 months to 5 years old and hospitalized with 1 or more of the following signs or symptoms that concern for lower respiratory tract infection (LRTI): accessory muscle use, chest pain, cough, crepitation or rales, decreased breath sound, dyspnea, prolonged expiration, wheezing.

MMBV, MeMed BV.

**Methods:**

Retrospective pragmatic study of MMBV implementation at a single medical center without specific AMS education. Children 3 months to 5 years old hospitalized with LRTI symptoms were included. In the non-interventional arm (2014-2017) MMBV results were not available in time to impact antibiotic decisions. In the interventional arm (2021-2022) MMBV results were rapidly available. Patients were assigned into discharge diagnosis classes: bronchiolitis, community acquired pneumonia (CAP), viral diagnosis and other diagnosis. Antibiotic administration was compared between arms per diagnosis class.

The relative reduction in antibiotic administration in the interventional arm versus the non-interventional arm according to discharge diagnosis class

**Figure:**
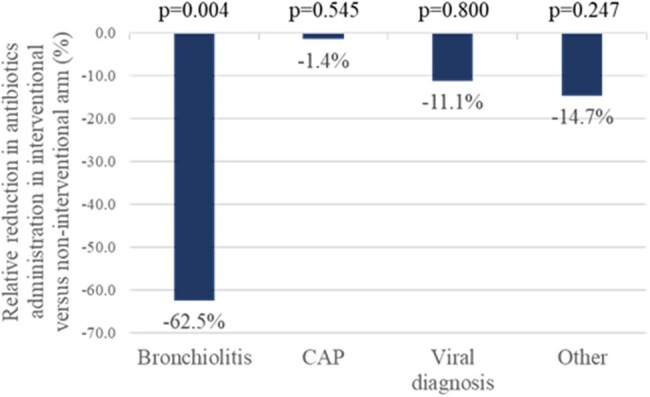

There was a relative reduction in antibiotic administration in the interventional arm relative to the non-interventional arm from 60.0% (12/20) to 22.5% (9/40) for bronchiolitis; from 98.7% (74/75) to 97.3% (71/73) for CAP; from 15.0% (12/80) to 13.3% (6/45) for viral diagnosis; and from 60.7% (51/84) to 51.8% (43/83) for other diagnosis.

P-values were calculated using Richardson’s method. CAP, community-acquired pneumonia.

**Results:**

Age, sex and MMBV results were similar across arms. Viral MMBV rates were high (39%-90%) across all diagnosis classes in the non-interventional (n=259) and interventional (n=241) arms. A significant relative reduction in antibiotic administration of 62.5% (p=0.004) was attained in patients discharged with bronchiolitis without impacting length of stay (p=0.530). Non-significant reductions in antibiotic administration were observed for CAP, viral diagnosis and other diagnosis.

**Conclusion:**

MMBV availability is associated with judicious antibiotic administration to children hospitalized with bronchiolitis without AMS education. Combining MMBV introduction with AMS education is required to reduce potentially unwarranted antibiotics in children hospitalized with CAP.

**Disclosures:**

All Authors: No reported disclosures

